# The composition, leaching, and sorption behavior of some alternative sources of phosphorus for soils

**DOI:** 10.1007/s13280-014-0615-7

**Published:** 2015-02-15

**Authors:** Marc I. Stutter

**Affiliations:** The James Hutton Institute, Aberdeen, AB15 8QH UK

**Keywords:** Soil amendments, Phosphorus, Nutrient compositions, Leaching, P availability

## Abstract

Concerns about the sustainability of inorganic fertilizers necessitate the characterization of alternative P source materials for agronomic P-efficiencies and P losses via leaching. Firstly, this study examined nutrient compositions including P speciation of seven soil amendments: sewage sludge (SS), anaerobic digestate (AD), green compost (GC), food waste compost (FWC), chicken manure (CM), biochar, and seaweed. Secondly, soil P leaching and availability was studied on a subset of four materials (SS, AD, GC, and CM). Sorption of extracts onto columns of a test soil showed strong P retention for SS and compost, but weak P sorption for CM and especially AD, suggesting short-term leaching risks for soil applied AD. Limited P desorption with water or citrate indicated sorbed P was strongly fixed, potentially limiting crop availability. These data indicate that variation in P forms and environmental behavior should be understood to maximize P usage, but minimize leaching and soil P accumulation. Hence, different alternative P source materials need differing recommendations for their agronomic management.

**Electronic supplementary material:**

The online version of this article (doi:10.1007/s13280-014-0615-7) contains supplementary material, which is available to authorized users.

## Introduction

The current global phosphorus cycle is inefficient with regard to fertilizer P usage and reuse of P-containing wastes, leading to global depletion of high-quality mineral phosphate stocks (Cordell et al. 2011). A European-level budget (per capita values for the EU-15) showed that for an average 4.7 kg P year^−1^ net consumption, only 0.8 kg P year^−1^ is currently recycled (Ott and Rechberger 2012). There is a need to redistribute secondary P (i.e., non-mineral and biosphere P) back into agricultural systems to tighten P cycles (Elser 2012). Secondary P resources comprise natural organic and waste materials utilized for their fertilizer properties including human and animal excreta either combined or separated (e.g., source-separated urine, Maurer et al. 2006), agricultural, municipal and domestic wastes via composting or post-energy recovery methods such as anaerobic digestion, and soil improvers like biochar (Schnell et al. 2012).

Sewage sludge (SS) has long been applied to land as raw or processed sludges, for example, struvite precipitation (Muster et al. 2013) or thermal ashing P recovery (Verstraete et al. 2009). The EU-27 produces 10 million tons of dry solids annually, with 40 % applied to agricultural land (Roig et al. 2012). The wastewater industry is undergoing a transition toward resource recovery rather than processing to gain compliance for environmental discharges (Verstraete et al. 2009; Mitchell et al. 2012), driven both by raw material economics and tightening legislation for landfill disposal and water pollution (Sartorius et al. 2012). Realization of the embodied energy value of organic wastes is leading to increasing availability for nutrient recovery of anaerobic digestates (AD) after biogas energy production from agricultural or food waste (Mehta and Batstone 2013). Other P-containing materials have long been traditionally used, but variably studied. Manures have been the subject of numerous evaluations, but others such as seaweed considerably less so.

There is a need for improved characterization of alternative P sources in terms of their P availability and environmental interactions. Studies often address contaminant issues in soil amendments (Diaz-Cruz et al. 2009; Linderholm et al. 2012). However, Toor et al. (2006) proposed better knowledge on P solubility, partitioning and bioavailability are necessary to balance soil amendment fertilizer potential with protecting the environment from negative consequences of soil P accumulation, erosion losses, or leaching. Well-documented mineralization rates for composts and manures focus on N transformations since legislation sets agronomic loadings by N (Jin et al. 2011; Zarabi and Jalali 2013). A review on compost nutrient cycling by Prasad (2013) suggests that knowledge on P mineralization and leaching following compost amendments is limited but that application rates ought to be set on P, not N, availability.

Quantification of different P species by ^31^P NMR has been undertaken for SS (Frossard et al. 2002; Escudey et al. 2004; Peng et al. 2010), manure (Lehmann et al. 2005), and compost (Galvez-Sola et al. 2010) to inform on P speciation. Stutter et al. (2012) argued that current agronomic P inefficiencies have led to accumulating soil P in complexed forms that current crops cannot access. So what if the P forms in these potential alternative P sources largely remain unavailable to crops due to P speciation and/or fixation in soils? This scenario may lead to further system inefficiencies and eventual increased P transfer to waters, as postulated by Escudey et al. (2004), and requires technological developments to manage.

To date there have been limited studies using consistent methodologies across a range of alternative P supply materials to evaluate compositions, environmental interactions, and support field-scale agronomic trials data. The hypothesis of this study was that large variation in P and organic matter compositions between alternative fertilizer materials controls different environmental P behaviors and necessitates balancing beneficial aspects such as P availability against negative aspects such as P leaching. The approach to testing this aimed to (1) evaluate nutrient compositions for a wider set of seven alternative P source materials, then (2) on a subset of four materials (selected on the basis of variation in composition and likely product availability) to evaluate P leaching and sorption using column and batch studies.

## Materials and methods

Methods are described here briefly with full details given in the Electronic Supplementary Materials methods section.

### Sample collection

SS was collected from a city municipal processing plant (~250 000 p.e.) operating the Cambi thermo-hydrolysis process, that induces high temperatures and pressures using steam to disintegrate sludges for enhanced biogas recovery by anaerobic digestion (patent Cambi AS, Norway). The final dewatered sludge is sold as an agricultural amendment. Anaerobic digestate (AD) liquor came from an on-farm biogas plant processing a mixture of slurry and abattoir waste. Two types of compost were collected from a large commercial plant. Green compost (GC) comprises municipal and local authority green waste and is a certified product under the UK PAS100 scheme. Premium compost is the green compost amended with food waste compost (FWC). Commercial pelletized and dried chicken manure (CM) was sourced from a local garden center. Biochar comprised commercially sourced pyrolysis-treated softwood. Seaweed was washed and dried marine macroalgae of species *Fucus vesiculosus* and is representative of the dominant P fertilizer material in use in small-scale agriculture by Atlantic fringe communities even up to modern times.

### Analytical methods

Materials were air-dried (30 °C) for analysis. Carbon and nitrogen contents were determined by combustion chromatography (Thermo Finnigan Flas EA 1112, Waltham, USA) and Total P by a NaOH fusion method (Smith and Bain 1982). Analysis of extraction solutions was done by ICP-OES for metals (Optima 5300DV, Perkin Elmer) and by automated colorimetric methods (Skalar San++, Breda, the Netherlands) for nutrients DOC, N, and P. The analytically defined fractionation of P yielded total dissolved P (TDP), dissolved reactive P (MRP; principally PO_4_-P), and dissolved unreactive P (MUP; principally organically complexed).

Solution Phase ^31^P NMR was performed on extracts (0.25 M NaOH and 0.05 M EDTA at 1:20 w/v ratio; Turner et al. 2003) of materials. Spectra were acquired using an Avance 500 II instrument (Bruker, Germany) employing a pulse delay of 2 s and a 90° pulse angle and using an internal standard of methylene diphosphonic acid (MDPA; Bedrock et al. 1994) for quantification.

Identification of mineral phases using X-ray powder diffraction (Siemens D5000 diffractometer) was undertaken where inorganic P dominated in SS and AD samples. Identification of minerals was made on background subtracted spectra against a library of standard pure mineral phases.

### Water extraction and column sorption experiments

Solution P extracts from a subset of four amendments (SS, AD, GC, and CM) were generated for use in subsequent column experiments. This selection of materials was made on the basis of (i) variation in compositional aspects of P speciation, total P, organic matter content, and (ii) current consumer uptake and future likely availability of the products for fertilizers. Water extractable P (WEP) is a common measure for assessing potential environmental P mobilization associated with soil amendments and for leaching studies (Kang et al. 2011). The extracts for the column leaching experiments were made by equilibrating 100 g each of air-dried SS, AD, CM, and GC materials with 4 L of 1 mM NaCl at room temperature on an end-over-end shaker for 16 h.

The subsets of four WEP solutions were sorbed onto duplicate glass columns (2.5 cm diameter, 6 cm length; Sigma, UK) repacked with a test soil (29 g air-dried) at 20 °C during saturated column flow experiments. The Strichen B soil (a Spodosol B horizon; described in the Electronic Supplementary Material) has previously been used as a test soil due to a strong sorption affinity for anions such as DOC and PO_4_^3−^ (Lumsdon 2004). From a background of 1 mM NaCl column, inflows were switched to the WEP solutions (1 mL min^−1^) and samples taken for 57–82 PVs over 22 h. Eluant samples were analyzed for P forms and UV absorbance. Sorption was studied at the pH and ionic conditions of the WEP solutions without further control. The background matrix of 1 mM NaCl approximated the ionic strength of rainwater, additionally minimizing effects due to varying ionic strength, as previously in Stutter et al. (2007, 2009).

### Column material recovery and citrate extraction

Following column P loading, one column from each of the replicate pairs was destructively sampled and a back-extraction performed to examine P availability from the packing soil using (a) water and (b) citrate (Na salt, extract concentration 50 μM) in 15 mM MES buffer (2-(*N*-morpholino)ethanesulfonic acid) at pH 5.5 according to George et al. (2007).

### Statistical analyses

Data were evaluated in Minitab (v.16). Relationships between analytical parameters were assessed using linear regression following normality testing (Andersen–Darling tests) and *t* tests used to determine differences between triplicate replicates of desorption with water or citrate. In Table 4, relative standard deviation is used to portray uncertainties in duplicate column leaching parameters, or triplicate back extractions from column soils. Column eluant breakthroughs were evaluated graphically. NMR spectra were processed using MestReNova (v.9.1.0).

## Results

Compositions of the seven amendments are shown in Table 1 (for general properties) and Table 2 (NaOH–EDTA extracts). The largest (95 %) moisture content was for AD followed by SS, FWC, and GC. The seaweed sample was received air-dried and an assumed 80 % moisture content was used. CM and biochar were dried during the production processes. The order of total P by NaOH fusion digest (air-dried basis; Table 1) was AD > SS > CM > FWC > seaweed > GC > biochar. The greatest C content was for biochar (573 mg C kg^−1^) and smallest for FWC (257 mg C kg^−1^) and for other materials between 300 and 400 mg C kg^−1^. Larger N contents for sludge, AD, and CM gave low C:N ratios all of 8. SS and AD had C:P ratios <30. FWC had a smaller C:N and C:P than GC.

**Table 1:** Origins and characteristics of samples (ordered by decreasing dry mass P content)

Sample	Origin	Process and material sources	Moisture content (g kg^−1^)	N (g kg^−1^)	C (g kg^−1^)	P (g kg^−1^)	C:N	C:P	P (kg tonne^−1^ original weight)
Sewage sludge (SS)	Municipal plant	Cambi-process, domestic sewage	72	39.6	321	20.8	8	15	5.8
Anaerobic digestate (AD)	Farm AD plant	Slurry and abattoir waste	95	43.4	366	14.7	8	25	0.7
Chicken manure (CM)	Garden center	Commercial dried, pelletized	<5	40.4	335	6.2	8	54	5.9
Food waste compost (FWC)	Commercial plant	Green plus food waste	52	19.1	257	3.1	13	82	1.5
Seaweed (*Fucus vesiculosus*)	Scottish beach	Washed in freshwater, air-dried	80	26.9	367	2.8	14	131	0.6
Green compost (GC)	Commercial plant	PAS100 certified green waste source	40	13.5	381	2.7	28	141	1.6
Biochar	Commercial plant	Softwood pallets	<5	1.4	573	0.2	409	3723	0.1

**Table 2:** Compositions of NaOH–EDTA extracts (with extract P recovery compared with NaOH fusion total P). Concentrations in g kg^−1^ dry soil

Sample	TDP^a^	MRP	MUP	% MUP	Extract P recovery (%)^b^	Ca	Fe	K	Mg	Mn	P/(Fe + Mn) ratio^c^
Sewage sludge	20.32	17.80	2.53	12	98	26.69	0.65	0.72	0.54	0.50	17.7
Anaerobic digestate	12.59	10.60	1.99	16	85	20.86	1.99	32.65	1.23	0.04	6.2
Chicken manure	6.00	2.52	3.48	58	97	34.76	0.15	18.98	2.16	0.07	28.3
Food waste compost	2.93	1.96	0.96	33	94	30.12	0.61	8.52	0.90	0.15	3.9
Seaweed	2.28	1.08	1.19	52	81	9.44	0.07	26.47	5.08	0.24	7.5
Green compost	2.45	1.70	0.74	30	91	10.37	1.94	6.05	1.13	0.19	1.2
Biochar	0.04	0.03	0.01	34	25	0.53	0.18	0.19	0.05	0.02	0.2

^a^Total dissolved P (TDP) represents the sum of molybdate reactive P (MRP) and molybdate unreactive P (MUP)

^b^Extract P recovery compared with NaOH fusion total P (as in Table 1)

^c^P/(Fe + Mn) mass ratio for concentration of P relative to paramagnetic ions in assessing ^31^P NMR conditions

For NaOH–EDTA extracts, the order of TDP was sludge ≫ AD > CM > FWC > GC > seaweed ≫ biochar (Table 2). CM (58 %) and seaweed (52 %) had large contributions of organically complexed P in the extracts (MUP:TDP), with similar contributions (30–34 %) for both composts and biochar, but lower contributions for SS (12 %) and digestate (16 %). Concentrations of metals are given in Table 2. Concentrations of Fe were large in AD and GC, but small for other materials. Chicken manure, FWC, sludge, and AD had large Ca extract concentrations, with the high Ca:P ratios (>10) for biochar and FWC. Extracts of AD, seaweed, and CM had larger K than other materials.

**Fig. 1:**
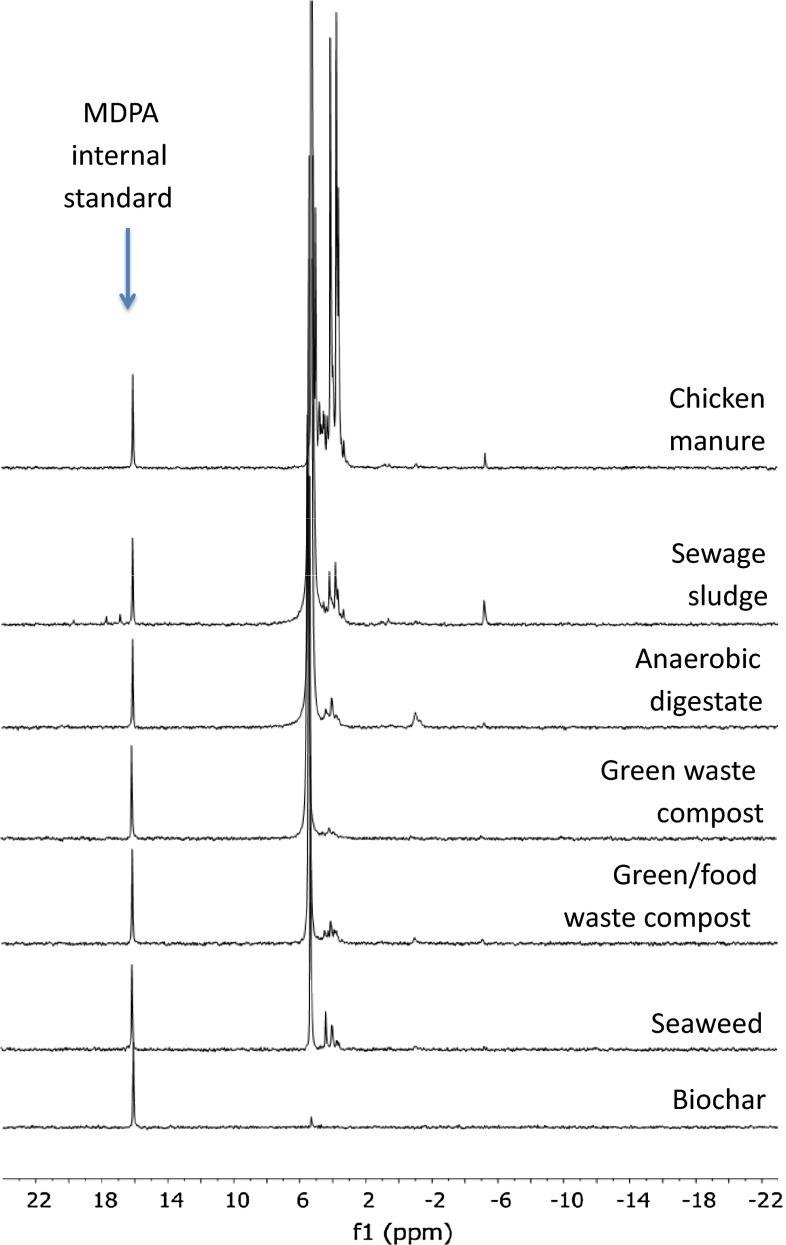
^31P^NMR spectra of the seven soil amendment materials. Spectra are common y scaled according to the peak height attributed to the fixed mass of MDPA (methylene diphosphonic acid; 16.8 p.p.m.) included in the NMR analysis as an internal standard to facilitate quantitative analysis

For quantification, the ^31^P NMR peaks were normalized to the internal MDPA standard (Table 3; Fig. 1). Extraction efficiencies (NaOH–EDTA TDP relative to NaOH fusion; Table 1) were appropriately large for quantitative NMR (except for biochar due to low P concentrations). In terms of confidence in the quantification procedure, a highly significant relationship was attained between the NaOH–EDTA extract TDP, and the sum of peak areas of all P signals normalized to the MDPA peak area (*P* signal area_norm_ = 0.0045 × TDP_NaOH–EDTA_ − 2.14; *R* ^*2*^ = 0.98, *p* < 0.001). Despite large organic matter contents, the dominant P form in most materials was inorganic orthophosphate P (ortho-*P*_*i*_), being 88–95 % of total P, except for seaweed (72 % ortho-*P*_*i*_) and CM (48 % ortho-*P*_*i*_). Concentrations of organically complexed P (*P*_o_) decreased CM > SS > AD > Seaweed > FWC > GC > biochar. CM had a strong monoester P signal (Fig. 1). Monoester P dominated *P*_o_ generally, except AD contained an appreciable amount of diester P and SS contained pyrophosphate P and some phosphonate. There was a significant relationship between the % *P*_o_ compounds to total P determined by NMR and the % MRP of TDP determined by colorimetry on extract solutions (*P*_o_ by NMR = 0.89 × %MRP − 11.2; *R* ^*2*^ = 0.79, *p* < 0.01).

**Table 3:** Characterisation of P compounds by ^31^P NMR reported as gP kg^−1^ dry soil. *ND* denotes not detected

Sample	Phosphonates	Polyphosphates	Inorg orthoP	Monoesters	Diesters	Pyrophosphate
NMR shifts (p.p.m.)	17.8, 20.6	20.5	6.2	6.0, 4.1	−0.2, −0.3	−4.2, −4.4
Sewage sludge	Trace	*ND*	19.36	0.87	Trace	0.09
Anaerobic digestate	*ND*	*ND*	11.68	0.68	0.23	Trace
Chicken manure	*ND*	*ND*	5.07	5.48	Trace	0.04
Food waste compost	*ND*	*ND*	2.58	0.34	Trace	Trace
Seaweed	*ND*	*ND*	1.64	0.63	Trace	Trace
Green compost	*ND*	*ND*	2.25	0.20	Trace	Trace
Biochar	*ND*	*ND*	0.04	*ND*	*ND*	*ND*

Despite large concentrations of ortho-P_*i*_ determined by ^31^P NMR in both SS and AD, the XRD analysis showed no identifiable crystalline mineral phosphate forms (Electronic Supplementary Material, Figs. S1, S2). Both samples contained quartz and calcite, with NaCl and KCl salts in the AD sample (corresponding to high Ca in both materials and K for AD in extracts; Table 3). Additionally, barite may indicate oil industry waste in SS.

Four air-dried materials were extracted into a 1 mM NaCl solution to determine water extractable P (WEP; Table 4a). The P solubility (% of total P) ranged from CM (18 %) > AD (5 %) > GC (3 %) > SS (0.3 %). In AD and CM, the WEP was dominantly molybdate unreactive (MUP; mainly organic P), with an indication of concentrated soluble dissolved organic matter (especially CM, shown by UV absorbance at 285 nm). Conversely, for GC and SS extracts, lower WEP concentrations comprised dominantly molybdate reactive P (MRP; mainly inorganic) with lower UV absorbance. Potentially leachable trace metals are shown in Table S1 (Electronic Supplementary Material). Water extract concentrations were low for Cd, Cr, and Ni but appreciable for Cu and Zn in CM.

**Table 4:** (a) Properties of water extracts used for sorption studies; (b) the column conditions and sorption of P onto soils; (c) the desorption of sorbed P from column soil packing under batch conditions with either water or 50 µM citrate. Error values shown are ±% relative standard deviations

Column feed solution	(a) Water extracts (mg L^−1^, except for pH and UV abs)					(b) Column P sorption					(c) Batch P desorption	
	UV_285_ (cm^−1^)	pH	TDP^a^	MRP	MUP	Max column breakthrough (*C*/*C*_0_)^b^	Column pore volumes leached^c^	MRP mass loading (mg)	% of P retained on soil	P mass sorbed (mg kg^−1^)	% P desorbed by water	% P desorbed by citrate
Sewage sludge	2.93	6.39	1.3	1	0.4	0.11 ± 5	65 ± 3	1.2 ± 2	90 ± 1	37	0.13 ± 62	0.04 ± 108
Anaerobic digestate	7.72	6.74	19.8	4.3	15.5	1.29 ± 9	82 ± 28	5.44 ± 3	4 ± 61	11	4.76 ± 21	6.24 ± 24
Green compost	2.79	6.92	1.7	1.7	<0.01	0.44 ± 2	73 ± 28	2.19 ± 7	69 ± 0	54	0.01 ± 173	0.13 ± 91
Chicken manure	26.86	6.88	28.2	7.3	21	0.98 ± 10	57 ± 8	8.25 ± 9	28 ± 18	82	0.03 ± 48	0.71 ± 56

^a^Total dissolved P (TDP) represents the sum of molybdate reactive P (MRP) and molybdate unreactive P (MUP)

^b^ *C/C*_*0*_ denotes the ratio of column eluant concentration divided by the inflow concentration

^c^Equivalent to: 1256, 1251, 1260, and 1137 mL leachate, respectively

Column breakthrough curves (BTCs) are shown in Fig. S3 and column parameters are shown in Table 4b. Leaching the WEP solutions onto columns of strongly P sorbing test soil for 57–82 column pore volumes (PV) gave final *C*/*C*_0_ values of 0.1 for SS, 0.4 for GC, with column P breakthrough (i.e., *C*/*C*_0_ ~ 1) only attained with feed solutions of AD and CM. Duplicate columns showed good general agreement in leaching behavior. Sewage WEP had the lowest MRP concentration (1 mg L^−1^) and attained the lowest *C*/*C*_0_ of 0.1. The BTC strongly plateaued after 10 PVs and the soil retained 90 % of the MRP loading. Digestate extract had 4 mg MRP L^−1^, attained *C*/*C*_0_ of 1.1 with a linear BTC and retention of 4 % of MRP loading. Green compost had extract 2 mg MRP L^−1^, attained *C*/*C*_0_ of 0.4 with a slightly curved BTC and retention of 69 % of MRP loading. CM WEP had 7.3 mg MRP L^−1^, high UV absorbance and attained MRP *C*/*C*_0_ of 1 with a linear BTC and 28 % retention of MRP loading. Using UV absorbance as a surrogate of DOC maximum, all columns attained maximum eluent concentrations for UV within 20 PVs at *C*/*C*_0_ of 0.87 for SS, 0.91 for AD, 0.96 for GC, and 0.98 for CM. Initial extract pH ranged from 6.4 (SS) to 6.9 (GC), although pH was not determined in eluents due to small sample volumes, and UV is sensitive to pH change.

Subsequent P desorption in batch systems (as a % of P-loaded onto the column packing soils) was only appreciable for AD where 5 and 6 % of P was desorbed using water and 50 mM citrate, respectively. This was despite the AD WEP loading (as a product of low overall % retention) giving the lowest overall mass of P sorbed onto the test soil. In all other cases, P desorption was ≪1 % of extract P sorbed. Low P concentrations gave large errors in replicates (*n* = 3), and no differences between desorption with citrate relative to water were significant at the *p* < 0.05 level using *t* tests.

## Discussion

### Organic amendment P compositions and availability

The greatest total P content in the present study was for SS (21 g P kg^−1^ dry mass), comparable to 27 ± 9 (to a maximum 55) g P kg^−1^ for sludges from 48 Swedish wastewater works (Eriksson 2001). Sludge compositions (Table 1) were similar in terms of dry matter, OM, C/N, total N, and P to those (also processed by anaerobic digestion) documented by Roig et al. (2012). Peng et al. (2010) reported solution ^31^P NMR data for sludges from 13 Chinese urban wastewater works and suggested that spectroscopic P-speciation studies complimented enzyme P studies in looking at potential bioavailability of *P*_o_ and *P*_*i*_. These authors showed sludge P species compositions comprised primarily inorganic ortho-phosphates with appreciable orthophosphate monoesters, similar to the current study (Table 3). Additional these authors observed minor contributions of the more labile* P*_o_ forms pyrophosphates and diesters in several samples. The current evidence of a dominance of *P*_*i*_ by solution NMR and MRP labile solution P in water and NaOH–EDTA extracts suggests high ‘potentially available’ P in SS that could be realized according to the extent of interactions with receiving soils.

Characterization is needed to inform appropriate utilization of the growing quantities of the by-product of biogas generation (anaerobic digestate; AD). Möller and Müller (2012) in a comprehensive review of AD in Germany reported large concentration ranges for dry matter (DM, 1.5–13 %), total N (3–14 % of DM), total C (36–45 % of DM), and total P (0.6–1.7 % of DM), reflecting a wide range in AD feedstocks. The AD in the present study from farm slurry and abattoir feedstock (Table 1) had nutrient contents at the low end of these ranges, but a large C:N ratio. The AD P species were dominated by inorganic orthophosphate P with minor contributions of monoesters and diesters (6 and 2 % of total P). The extracted NaOH–EDTA P was dominated (84 %) by MRP, but water solubilised P was dominated (80 %) by dissolved molybdate unreactive P (MUP), possibly influenced by soluble diester P. Möller and Müller (2012) summarized the transformations of nutrients during the AD process as a reduction in DM, OM, and C:N ratio, and found no change in total N but an increasing dominance of ammonium. For P, a loss in total P was attributed to increased pH, mineralization, and precipitation reactions involving Ca- and Mg- phosphates (e.g., struvite).Mehta and Batstone (2013) reported <10 % of total P in AD to be soluble, yet completely extracted in citrate, and they concluded P released by digestion was bound to inorganic compounds or sorbed onto solid surfaces. Although precipitation reactions limit *P*_*i*_ solubility, no crystalline P minerals were observed in the current dried sample (Fig. S2). In this study, AD liquor was air-dried and then re-extracted into water. However, bulky organic amendments can be considered not economically viable to transport in their raw state. Drying of AD now occurs in 1 % of German biogas plants (Möller and Müller 2012).

Total P contents of the composts agree with those reported by Frossard et al. (2002) for 15 alkaline composts (urban, garden, and farm manure substrates) of mean 3.4 (range 2.1–7.2) g P kg^−1^ dry matter. In the present study, FWC has greater P (and N) content but lower C than GC (Table 1). Resulting low C:P and C:N suggest greater N and P mineralization and potential mobilization when FWC is added to soils. Phosphorus species were similar between the composts with slightly greater monoester *P*_o_ forms in FWC (11 %) than GC (8 %; Table 3). Although NaOH–EDTA P compositions had an appreciable MUP content (30 and 33 % for GC and FWC, respectively), the water soluble P (Table 4; determined for GC only) was exclusively MRP. Frossard et al. (2002) concluded that 30–50 % of *P*_*i*_ in composts was rapidly plant available, with another 30–50 % of *P*_*i*_ formed of Ca-precipitates, although they urged caution that air-drying of materials could have increased P mineralization and hence *P*_*i*_ quantification.

CM had the third largest P (and N) content (after SS and AD; Table 1), but the greatest contribution of monoester *P*_o_ (Table 3) and a corresponding large fraction of MUP in NaOH–EDTA (Table 2) and water extracts (Table 4a). Many studies have used ^31^P NMR analysis to examine the P forms in manures (Peirce et al. 2013 and references therein). Often the specific form of inositol hexaphosphate dominates monoesters as it is not broken down in the gut of non-ruminants. Peirce et al. (2013) found that monoester P contributed about half of the P total in fresh manures, similar to the current study. These authors studied how monoester to inorganic orthophosphate ratios of chicken manures subsequently declined following the on-farm practice of manure stockpiling over increasing times, similar to observed mineralization of monoesters in soils.

There has been considerable interest in biochar as a soil conditioner and nutrient source. Although compositions vary greatly, it has been reported that 10–60 % of P is leachable from wood-derived biochar (Muckherjee and Zimmerman 2013). However, the pine-derived commercial biochar used in the present study had very small N and P contents (Table 1), with P speciation not resolvable by NMR. Large concentrations of Ca and Fe in the NaOH–EDTA extracts suggest potential for a high degree of interaction of P with bridging cations with the implication of P being more strongly bound. On the other hand, high C contents may lead to high DOC solubility and competitive sorption to maintain solubility of any small amount of P leached.

Natural materials such as seaweed have been used historically as soil amendments but lack current scientific evaluation of their P contents and speciation. Haslam and Hopkins (1996) focused on soil conditioning properties, reporting that kelp-amended soils had greater aggregate stability and enhanced microbial respiration and N mineralization rates at moderate applications. The current study showed monoesters contributed a quarter of total P with overall P contents similar to composts. Seaweed has potential as a P alternative given the abundant potential for supply globally, either directly or following use as an AD feedstock. Table 2 shows high K and Mg content which will add benefits when applied to the soil. However, investigations should be made of the consequences of associated marine-derived metal contaminant loading to soils (such as As).

A short pulse delay time (2 s) was used in NMR analysis. This parameter is important for attaining quantitative spectra, for example, McDowell et al. (2006) showed for soils that short delay times bias the results in favor of nuclei such as orthophosphate that relaxes quickly, and hence apparent diesters’ concentrations decrease. The time of relaxation is enhanced in the presence of Fe and Mn. Ratios of P/(Fe + Mn) in Table 2 show that under-representation of diesters may have been an issue for SS and CM.

### Potential leaching behavior of P from use of amendments

Solubility is a factor in crop availability of P species, but P leaching represents a concern on application or accumulation of P-rich organic materials in soils. In agronomic applications, such alternative fertilizer materials would be applied to soils at standard P loading rates. Hence, an improved future approach to leaching behavior may utilize fixed P mass: extract volumes (as opposed to the fixed total mass: volume approach used here) as preparation for soil leaching experiments. However, the WEP concentration order differed greatly from that of source material P contents. The WEP for CM and AD showed high P solubility but dominated by MUP (Table 4), which may be related to the organic P forms evident by NMR of monoester P for CM and diester P for AD, with the latter generally highly soluble (Table 3). Conversely, SS and GC (with NMR showing P dominated by inorganic orthophosphate; Table 3) had relatively insoluble P, dominated by MRP and exhibited strong sorption of these dilute P extracts onto the test soil (Table 4). Sorption interactions between phosphate anions and Fe, Al surface complexes (that dominate sorption in such acid Spodosols) are affected by solution pH and sorption competition with dissolved organic matter. The pH of the water extracts occupied a narrow range (pH 6.4–6.9), being relatively neutral compared with the soil pH of 4.7 (in CaCl_2_). So pH differences between amendment extracts would not explain sorption behavior. It was not possible to measure the DOC concentration due to analytical issues with excessive dilutions but UV absorbance (UV_abs_) was large for CM indicative of soluble aromatic humic substances. Competitive sorption between organic matter and P anions (Kang et al. 2011) would be expected to explain limited sorption of 28 % of extracted manure P onto the soil. However, the most limited (4 %) sorption of extracted AD P occurred at much smaller UV_abs_ and suggested that the P speciation limited interaction of AD WEP with Fe and Al surfaces in such soils.

Precipitation reactions may also impact on P solubility. Frossard et al. (2002) found the presence of sparingly soluble, but poorly crystallized Ca–P forms (apatites, octocalcium phosphates) in composts, which they proposed were related to Ca content but also to the presence of organic substances inhibiting crystallization. The solubility of inorganic P forms is governed by associations with organic matter and other cations forming complexes or mineral phases. The molar Ca:P ratios (derived from Table 2) were close to unity but no crystalline phosphate minerals were observed by XRD in SS or AD, only calcite. Previously Frossard et al. (2002) observed a complex mixture of octocalcium phosphates, apatites, with additionally monetite in anaerobically digested sludge.

Column systems were chosen rather than batch systems to simulate field P leaching risk since columns provide a representation of breakthrough gained from multiple samples in time and integrating physico-chemical aspects of leaching behavior (Stutter et al. 2007). Water extracts had different P concentrations and using columns experienced different loadings of P. The breakthrough curves (Fig. S3) show *C*/*C*_0_ = 1 attained for AD and CM. Extrapolating these simulations to field conditions would approximate to P breakthrough for AD and CM in ~2000 mm rain (1–2 years amount) for 6 cm soil depth. Attainment of equilibria for input and output concentrations suggests saturation of the surfaces but this occurred at very different total P loadings of 11 and 82 mg P kg^−1^ soil for AD and CM extracts, respectively (Table 4). At the end of the column loading, *C*/*C*_0_ concentrations of 0.1 and 0.4 had been attained for SS and GC, respectively. Hence, P sorption for these extracts was capable of continuing beyond the final loadings of 37 and 54 mg P kg^−1^ soil. Due to the complex set-up and large number of samples, generated column experiments are seldom replicated, yet the results here showed close agreement between the column duplicates in terms of the breakthrough plots (Fig. S3), *C*/*C*_0_, and total P mass loadings (<10 % relative standard deviations; Table 4b).

The batch P desorption of soil recovered from the P-loaded columns evaluated reversibility of P fixation. One column of each pair was destructively sampled, once similarities in loadings and breakthrough of the duplicate columns were confirmed. The only appreciable desorption with water occurred for AD (5 % of sorbed P re-released), with limited additional release using 50 µM citrate (Table 4). Taken together these column sorption and desorption, experiments suggest that SS and GC would likely leach relatively small concentrations of MRP that is strongly and irreversibly sorbed by strongly reactive soils (such as Spodosols with large reactive Fe, Al concentrations). CM represents a greater leaching risk for soluble P (principally MUP) and has weaker interactions with soils due in part to high DOC concentrations. AD gave the greatest P leaching risk with a moderately high solubility and very limited soil sorption. Hence, these four alternative P amendments have very different P leaching risks and especially for AD would need strategies in place to minimize leaching, such as limiting applications to periods of crop growth. There are caveats to these findings such as the pre-treatment of drying and resolubilizing materials such as the AD. Such drying and rewetting may be expected to increase pools of soluble P, for example, associated with lysed microbial cells (Blackwell et al. 2009). However, drying pre-treatments may be part of necessary handling for bulky organic amendments.

Negative consequences of leaching have been long recognized for manures. Kang et al. (2011) examined P leaching from columns of sandy soils under various manure fertilizer and inorganic P additions. Recovery of MRP relative to added water extractable P was 60 % for inorganic P, but >100 % for manures, and these authors explained this in terms of *P*_o_ mineralization and competitive sorption of DOC. There is a need to extend leaching behavior studies to new waste streams such as AD. Möller and Müller (2012) argued that although German biogas plants produce 66 million m^3^ of digestate (74 Gg P), ‘no studies about the effects of AD on P losses via leaching and runoff after field application could be found.’ Walsh et al. (2012) examined additions of chemical fertilizer, undigested slurry, and AD liquor (from a slurry feedstock similar to the material in the present study) at matched N additions onto grassland and measured soil solutions for 10 weeks post application. They found that AD and slurry gave significantly lower NO_3_ and NH_4_ leaching, but did not measure P leaching.

## Conclusions

Alternative P sources in agriculture need to be characterized for P leaching and availability to maintain agronomic productivity but control environmental P losses to waters. The seven materials tested had two orders of magnitude difference in dry mass P content and differed in P speciation, C:N and C:P stoichiometry, trace and major metal contents, and physical handling properties in raw states. These variations in nutrient contents, forms, and stoichiometry have consequences for utilization of materials to realize the P content for crop growth in terms of biogeochemical cycling in soils through microbial decomposition, chemical solubility, and root uptake pathways. A subset of four air-dried materials selected on likelihood of fertilizer substitution revealed varying P solubility in artificial rainwater in terms of molybdate reactive (principally inorganic P) and unreactive solution P (principally organic P), the latter corresponding with source material organic P forms observed by ^31^P NMR. Interactions with an iron-rich test soil during column flow simulations of field leaching showed SS and compost were strongly sorbed, yet CM and especially AD were weakly sorbed and exhibited P breakthroughs. Batch P desorption to water or citrate was limited but greatest for AD (<5 % of sorbed P). Hence, utilizing alternative ‘waste’ materials with different compositions as replacement P fertilizers will necessitate different management strategies to minimize P leaching risk and ensure availability for crop uptake by limiting competitive soil P fixation.

## Electronic supplementary material

Below is the link to the electronic supplementary material.

Supplementary material 1 (PDF 367 kb)
