# Supplementary material for: The composition, leaching, and sorption behavior of some alternative sources of phosphorus for soils
Source: Ambio. 2015 Feb 15;44(Suppl 2):207–16. doi: 10.1007/s13280-014-0615-7 (PMC4329151; doi:10.1007/s13280-014-0615-7)
Supplement: Supplementary file 1 — Supplementary material 1 (PDF 367 kb) [file 13280_2014_615_MOESM1_ESM.pdf]

AMBIO

Electronic Supplementary Material

This supplementary material has not been peer reviewed.

Title: **The composition, leaching and sorption behaviour of some alternative sources of phosphorus for soils**

Author: Marc I. Stutter

The James Hutton Institute, Aberdeen, AB15 8QH, UK

Supplementary Material: Material and Methods details

Solid phase analysis Carbon and nitrogen contents were determined by combustion chromatography of ~2 mg ball-milled subsamples (Thermo Finnigan Flas EA 1112, Waltham, USA). Total P was determined by a NaOH fusion method (Smith and Bain 1982) comprising ignition of 0.1 g samples with solid NaOH, titrating the melt to pH7 with 20% H_2SO_4 and subsequent P analysis by ICP-OES, with CN and CP ratios on a mass/mass basis. Analysis of extraction solutions (as detailed below) was done by ICP-OES for metals (Optima 5300DV, Perkin Elmer) and by automated colorimetric methods (Skalar San++, Breda, the Netherlands) for nutrients DOC, N and P. Total dissolved P (TDP) was determined on filtrates following an automated heated persulphate digestion method and dissolved reactive P (MRP) without digestion, both using the molybdate reaction. Dissolved unreactive P (MUP; principally organically-complexed) was the difference TDP-MRP. The analytically defined fractions MRP and MUP equate approximately to PO_4-P and dissolved organically-complexed P (DOP), although there remains some uncertainties as to extent of hydrolysis of some DOP forms by the molybdate chemistry. The <0.45 µm filtration cut-off generally used for analysis meant that significant colloidal fractions could be present if dispersed by the extract matrix and many of these may be sufficiently labile to react with the molybdate chemistry.

Solution Phase ^{31}P NMR Air-dried materials were extracted with a solution containing 0.25 M NaOH and 0.05 M EDTA at a 1:20 w/v ratio (Turner et al. 2003). The extract was subsampled for determination of the total P concentration, and remainder of the extract freeze-dried. Immediately prior to NMR analyses 100 mg of freeze-dried material was dissolved into 1 ml of 1M NaOH containing 10% v:v D_2O for frequency locking (monitoring and correcting for 'drift' in the magnetic field). The solution was centrifuged and spectra were acquired using an Avance 500 II instrument (Bruker, Germany) employing a pulse-delay of 2 seconds and a 90° pulse angle. Quantitative data were obtained by using an internal standard comprising a fixed mass of a P compound non-native to soils (methylene diphosphonic acid; MDPA) in a sealed glass capillary included within the NMR sample tube (Bedrock et al. 1994).

X-ray Powder Diffraction Analysis The ^{31}P NMR revealed large concentrations of inorganic orthosphosphates in SS and AD samples and additional characterisation of these materials only was made by qualititative X-ray powder diffraction. Samples were prepared by McCrone milling followed by spray drying and run using cobalt radiation on a Siemens D5000 difractometer from 2-70 degrees two-theta, counting for 2 seconds per 0.02 degree step. Identification of minerals was made on background subtracted spectra against a library of standard pure mineral phases.

Water Extraction and Column Sorption Experiments Solution P extracts from a subset of four amendments (SS, AD, GC, CM) were generated for use in subsequent column experiments. A dilute salt matrix (1mM NaCl) simulating rainwater or dilute soil was used for P solution preparation, although this is hereby referred to as WEP. The extracts for the column leaching experiments were made by equilibrating 100 g each of air-dried SS, AD, CM and GC materials with 4 L of 1mM NaCl at room temperature on an end-over end shaker for 16 hours.

The subset of four WEP solutions were sorbed onto duplicate repacked columns of a test soil (described below) at 20°C during column flow experiments. The soil (29g air-dried, <2 mm sieved soil) was packed into glass chromatography columns (2.5cm diameter, 6 cm length; Sigma, UK). The columns were wetted slowly from the base upwards to ensure saturation. The initial equilibration was with 1mM NaCl to attain constant flow (1mL minute^{-1}), then samples were collected prior to, and at 15 to 60 minute intervals after, switching inflows to the WEP solutions (maintaining saturation). Column pore volumes (PV) ranged from 13 to 20 mL and leaching continued for 57 to 82 PVs over 22 hours. Column leachates were analysed colorimetrically for MRP and TDP. The columns were sealed at both ends with flow adaptors containing PTFE 20 µm filters and as samples were not filtered further prior to analysis the definition of dissolved P forms used here comprises some colloidal forms. UV absorbance (285 nm) was used as a surrogate for DOC concentration in the solutions due to DOC colorimetry problems due to high dilutions required. Thus sorption was studied at the pH and ionic conditions of the WEP solutions without further control and simulated

amendment applications to soils with rainfall induced leaching. Small soil columns were chosen for practical reasons in simulating field P leaching risk due to the strong P sorption to soils and to scale down the volumes of leachate required to achieve possible P breakthough. The background matrix of 1mM NaCl for both extraction of the organic materials and column preconditioning was chosen as approximating the ionic strength of rainwater, additionally minimizing effects due to varying ionic strength, as previously in Stutter et al. (2007, 2009).

The Strichen B soil (a Spodosol B horizon, sandy loam texture) has previously been used as a test soil with a strong sorption affinity for anions such as DOC and $PO4^{3-}$ (Lumsdon 2004). It is an acid soil (pH 4.7), with concentrated acid ammonium oxalate extractable Fe and Al (28 g and 10 g kg^{-1}, respectively; indicative of Fe, Al oxyhydroxide surfaces) and small concentrations of Ca (<1 mmol kg^{-1} in 1M NH_4 acetate) and organic C (34 g kg^{-1}). The rationale for using this soil was that it is a non-agricultural soil from NE Scotland with low inherent P content and high P sorption potential and has been used as a test soil in P and C sorption studies (e.g. Lumsdon 2004).

Column material recovery and citrate extraction Following column P loading one column from each of the replicate pairs was destructively sampled and a back-extraction performed to examine P availability from the packing soil (without drying). Destructive sampling of one column was chosen once it had been confirmed that the P adsorption and total P loadings were similar between each pair of columns. The soil from each column was homogenized and triplicate samples of 2 g soil (dry mass equivalent) were extracted using (a) water and (b) citrate (Na salt, extract concentration 50µM) in the presence of 15 mM MES buffer (2-(N-morpholino)ethanesulfonic acid) adjusted to pH 5.5. The pH was buffered due to convention in plant-soil-rhizosphere studies (George et al. 2007). Extracts, following shaking at 20˚C for 16 hours, were filtered <0.45 µm prior to MRP analysis by molybdate colorimetry. Included were blanks of reagents without soils and of the original Strichen Bs soil that had not been exposed to the P leaching in the columns.

Supplementary Material: Figures and Tables

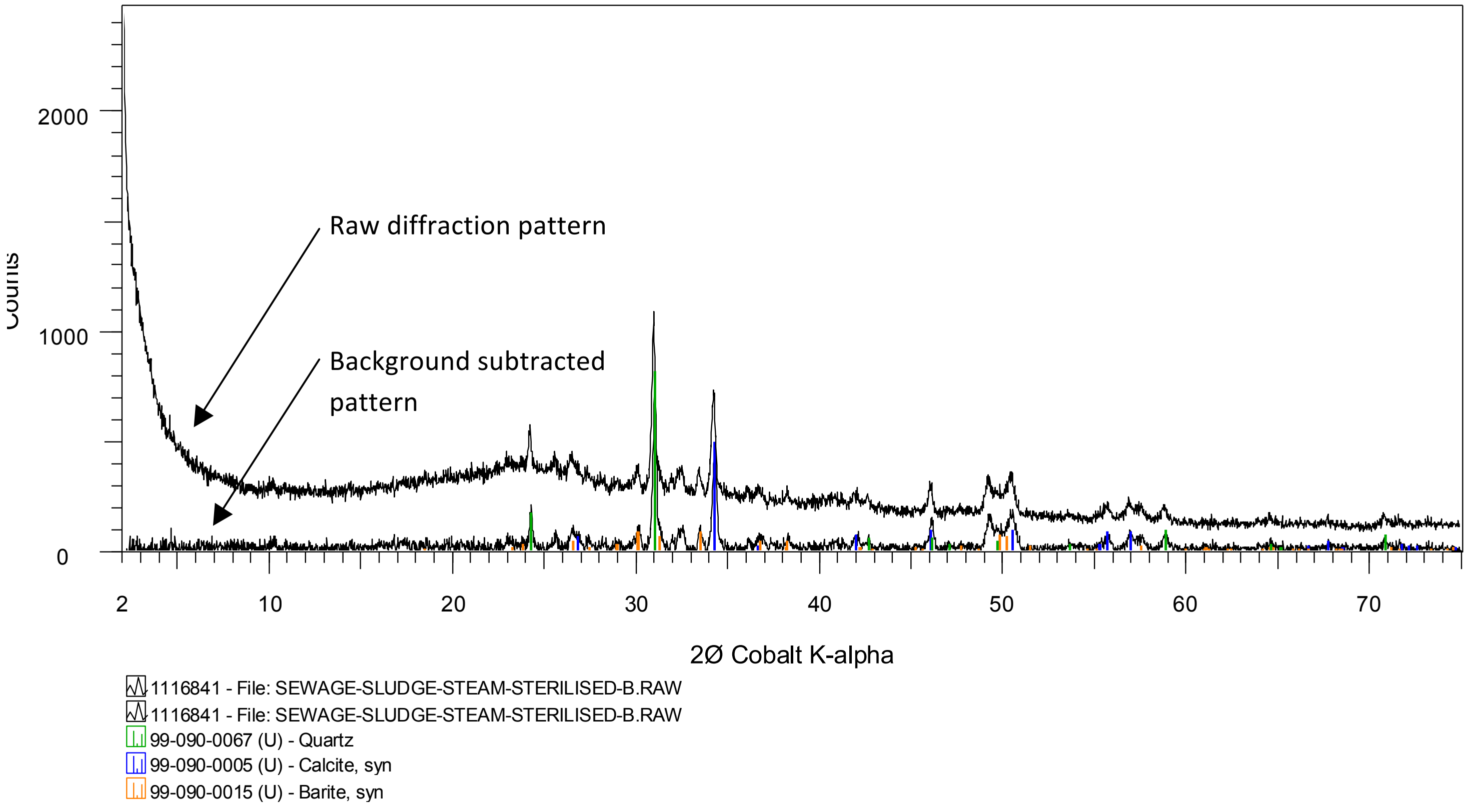

Fig. S1 X-ray powder diffraction mineralogical analysis of the dried sewage sludge raw material

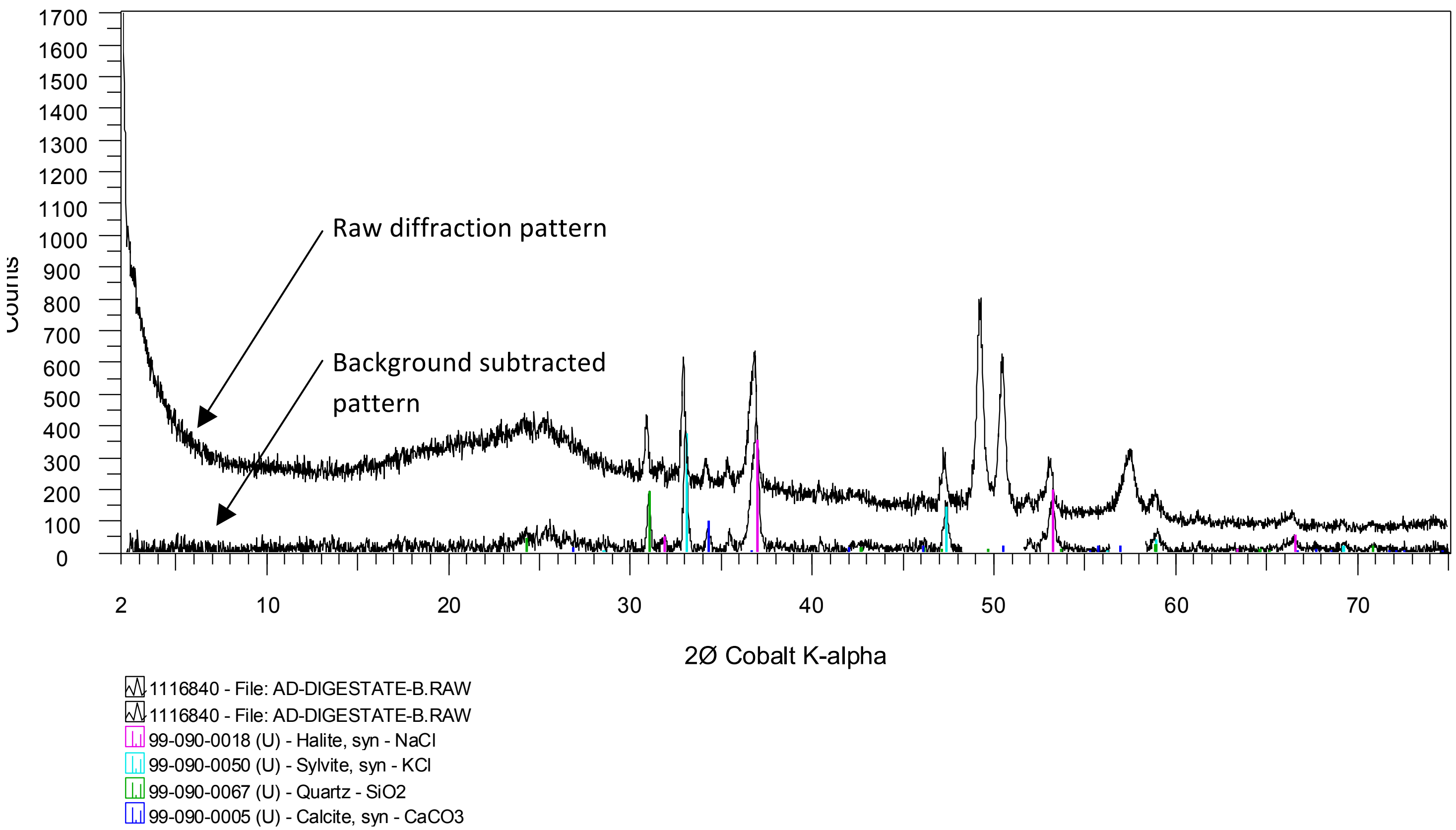

Fig. S2 X-ray powder diffraction mineralogical analysis of the dried anaerobic digestate raw material

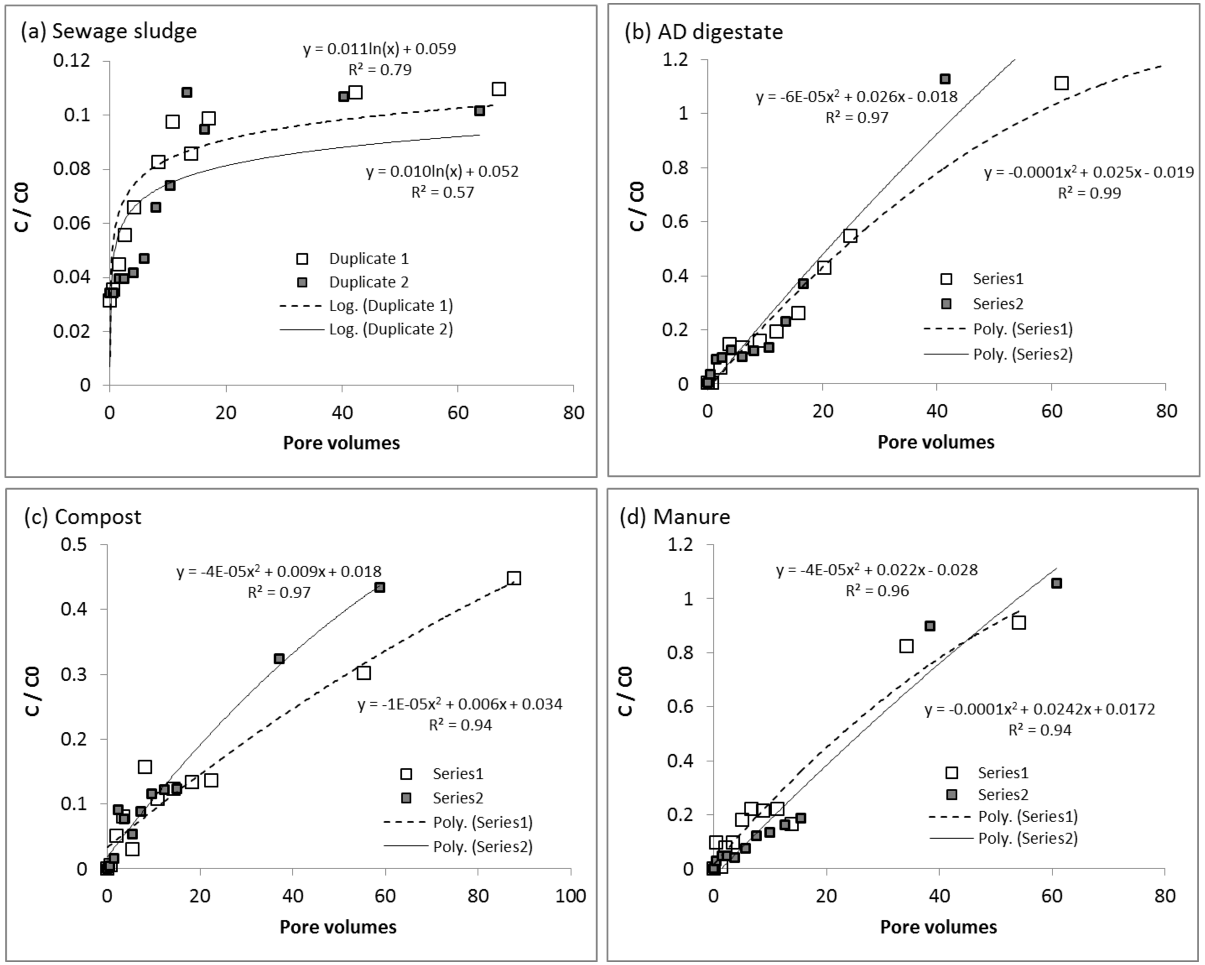

Fig. S3 Column breakthrough curves for eluent MRP concentrations (C) at time points relative to initial (C_0) at a given pore volume (PV). Trend lines are fitted on the basis of best fit principally to show general patterns and the consistency in leaching behaviour between duplicate columns

Table S1 Trace metals in water extracts used in column sorption experiments (concentrations as $\mu g\ L^{-1}$). Differences in detection limits and implied precision between filtrates arise due to varying dilution requirements associated with sample matrices. '<' denotes where a value of half analytical detection limit has been applied

Filtrates	As	Cd	Cr	Cu	Mo	Ni	Pb	Se	Zn
Sewage sludge	15	<0.4	<0.9	90	57	29	<2	150	17
Anaerobic digestate	26	<0.4	7.6	63	47	38	<2	250	81
Green compost	9	<0.8	<9	16	27	5	<20	50	31
Chicken manure	3	0.5	5.8	403	27	35	<2	30	428
